# Neighborhood-level deprivation and survival in lung cancer

**DOI:** 10.1186/s12885-024-12720-w

**Published:** 2024-08-06

**Authors:** Kathleen Kennedy, Ignacio Jusue-Torres, Ian D. Buller, Emily Rossi, Apurva Mallisetty, Kristen Rodgers, Beverly Lee, Martha Menchaca, Mary Pasquinelli, Ryan H. Nguyen, Frank Weinberg, Israel Rubinstein, James G. Herman, Malcolm Brock, Lawrence Feldman, Melinda C. Aldrich, Alicia Hulbert

**Affiliations:** 1 Department of Hematology Oncology, University of Illinois College of Medicine in Chicago, Chicago, IL USA; 2 Department of Neurologic Surgery, Mayo Clinic Health System, Eau Claire, WI USA; 3 Cancer Prevention Fellowship Program, Division of Cancer Prevention, National Cancer Institute, Rockville, MD USA; 4 Occupational and Environmental Epidemiology Branch, Division of Cancer Epidemiology and Genetics, National Cancer Institute, Rockville, MD USA; 5 Laboratory of Human Carcinogenesis, Center for Cancer Research, National Cancer Institute, Bethesda, MD USA; 6 Department of Surgery, Cancer Center, University of Illinois College of Medicine in Chicago, 909 South Wolcott Ave COMRB Suite 5140, Chicago, IL 60612 USA; 7 Department of Surgery, The Johns Hopkins University School of Medicine, Baltimore, MD USA; 8 Department of Radiology, University of Illinois College of Medicine in Chicago, Chicago, IL USA; 9 Division of Pulmonary, Critical Care, Sleep and Allergy, Department of Medicine, University of Illinois College of Medicine in Chicago, Chicago, IL USA; 10 Division of Research Services, Jesse Brown VA Medical Center, Chicago, IL USA; 11 Lung Cancer Program, University of Pittsburgh Cancer Institute, The Hillman Cancer Center, Pittsburgh, PA USA; 12 Sidney Kimmel Comprehensive Cancer Center at Johns Hopkins, Baltimore, MD USA; 13 Department of Medicine, Vanderbilt University Medical Center, Nashville, TN USA

**Keywords:** Lung cancer, Neighborhood-level deprivation, Disparities, Epigenetic, DNA methylation

## Abstract

**Background:**

Despite recent advances in lung cancer therapeutics and improving overall survival, disparities persist among socially disadvantaged populations. This study aims to determine the effects of neighborhood deprivation indices (NDI) on lung cancer mortality. This is a multicenter retrospective cohort study assessing the relationship between NDI and overall survival adjusted for age, disease stage, and DNA methylation among biopsy-proven lung cancer patients. State-specific NDI for each year of sample collection were computed at the U.S. census tract level and dichotomized into low- and high-deprivation.

**Results:**

A total of 173 non small lung cancer patients were included, with *n* = 85 (49%) and *n* = 88 (51%) in the low and high-deprivation groups, respectively. NDI was significantly higher among Black patients when compared with White patients (*p* = 0.003). There was a significant correlation between DNA methylation and stage for HOXA7, SOX17, ZFP42, HOXA9, CDO1 and TAC1. Only *HOXA7* DNA methylation was positively correlated with NDI. The high-deprivation group had a statistically significant shorter survival than the low-deprivation group (*p* = 0.02). After adjusting for age, race, stage, and DNA methylation status, belonging to the high-deprivation group was associated with higher mortality with a hazard ratio of 1.81 (95%CI: 1.03–3.19).

**Conclusions:**

Increased neighborhood-level deprivation may be associated with liquid biopsy DNA methylation, shorter survival, and increased mortality. Changes in health care policies that consider neighborhood-level indices of socioeconomic deprivation may enable a more equitable increase in lung cancer survival.

**Supplementary Information:**

The online version contains supplementary material available at 10.1186/s12885-024-12720-w.

## Background

Lung cancer is the leading cause of cancer-related mortality, accounting for almost 25% of all cancer-related deaths [1, 2]. With the implementation of lung cancer screening, targeted therapies, and immunotherapy, lung cancer survival has improved dramatically over the past couple of decades [3]. However, disparities in survival continue to persist among socially deprived and disadvantaged populations and hinder the full potential of modern medical advances to benefit all in society. While individual-level risk factors, such as tobacco use, have long been hypothesized to underlie this phenomenon, the role of broader socioeconomic factors on health outcomes is gaining recognition [3]. Neighborhood-level deprivation often encompasses geospatially aggregated (e.g., within U.S. census tracts or block groups) indices of employment, occupation, education, housing conditions, income, and wealth, which cumulatively can have profound impacts on health (7). Indeed, neighborhood-level factors can have detrimental effects on health outcomes, including 30-day hospital readmission rates, cardiovascular disease, type II diabetes, asthma, and chronic obstructive pulmonary disease [4, 5]. This association has also been demonstrated in lung cancer incidence, with one study finding that the odds of developing lung cancer increased by 66% in neighborhoods with the greatest deprivation [6, 7]. While the relationship between neighborhood-level deprivation and lung cancer mortality has been explored [8–10], the specific biological mechanism by which neighborhood conditions promote lung cancer progression remains uncertain. Epigenetic modifications are heritable changes in gene expression that occur without changes in the DNA sequence and can be influenced by many environmental factors [11, 12]. Acquired epigenetic changes promote initiation and progression of cancer by modulating gene expression, which plays a significant role in the initiation and progression of several types of cancer, including lung cancer [13, 14]. One of the most influential epigenetic changes involves methylation of CpG islands within the promoters of genes. Promoter hypermethylation of tumor suppressor genes leads to transcriptional silencing, which drives carcinogenesis. The impact of neighborhood-level deprivation on epigenomic gene expression and lung cancer mortality has yet to be explored.

This study aimed to assess the association between neighborhood-level deprivation and lung cancer mortality within the context of the U.S. health care system, as well as investigate its effect on the methylation status of six lung cancer tumor suppressor genes obtained from liquid biopsies. We hypothesized that patients with lung cancer and greater levels of neighborhood deprivation have a decreased overall survival and higher levels of tumor suppressor gene methylation.

## Methods

### Study design

This is a multicenter retrospective cohort study assessing the relationship of NDI with survival adjusted for age, stage, and DNA methylation among biopsy-proven lung cancer from two tertiary academic medical centers between 2008 and 2020. The reporting of this study conforms to the Strengthening the Reporting of Observational Studies in Epidemiology (STROBE) statement [15].

### Study population

Participants were referred for surgical resection based on a suspicious finding on chest CT. The inclusion criteria for this study comprised: (A) any adult age 30 or older with a diagnosis of non small cell lung cancer of any stage, either biopsy-proven or pathologically proven from a surgical specimen from surgery involving a lobectomy, pneumonectomy, or greater resection (stages were defined according to revised TNM guidelines classification criteria) [10]; (B) able to provide informed consent for this study. Exclusion criteria consisted of: (A) patients with other malignancies who preoperatively were incorrectly assumed to have primary lung cancer; (B) history of hereditary cancer; (C) radiotherapy or chemotherapy treatment had been given prior to surgical resection; (D) any patient < 30 years old; (E) pregnant patients. Clinical information collected included age at diagnosis, sex, race, tobacco use status (current vs. former vs. never), pack-year tobacco use history, tumor size, histology, stage at diagnosis, and residential address. This study was conducted in accordance with the Declaration of Helsinki. Institutional review board (IRB) approval was obtained prior to study initiation (IRB #2017–1286 and NA_00005998). Informed consent was obtained from all participants in the study.

### Neighborhood deprivation

Participants’ residential addresses at the sample collection were geocoded to U.S. census tracts using the U.S. Census Bureau geocoder and geographically linked to NDI values. State-specific (Illinois and Maryland tracts as the referent) NDI scores for each year between 2010 and 2020 were computed for U.S. census tracts using the “ndi” package in R [16, 17]. Messer et al. [18], previously defined and described the computation of NDI. Briefly, each census tract’s NDI value is its score from the first principal component of a Principal Component Analysis comprised of eight sociodemographic variables from the U.S. Census Bureau American Community Survey, including percent males in management, science, and arts occupation, percent of crowded housing, percent of households in poverty, percent of female-headed households with dependents, percent of households on public assistance, percent of households earning <$30,000 per year, percent earning less than a high school education, and percent unemployed [18]. Census tract-level NDI values were further categorized into quartiles, with the 4th quartile representing a census tract (i.e., neighborhood) with the highest deprivation and the 1st quartile representing a census tract (i.e., neighborhood) with the lowest deprivation (relative to all Illinois and Maryland census tracts). They were further dichotomized into a “low NDI” group defined as the 1st, 2nd, and 3rd quartiles and a “high NDI” group defined as the 4th quartile. The classification of high vs. low NDI was primarily based on maintaining a balanced sample size between groups to allow for robust statistical comparisons. We computed census tract-level NDI scores and quartiles per year (2010–2020) and geographically linked them to participant residential addresses for their year of collection. Participants collected in 2008 or 2009 were assigned 2010 census tract-level NDI scores and quartiles.

### DNA methylation

DNA extraction and isolation from plasma-based liquid biopsy samples were performed following the same methodology as previously described [8, 9]. ß-Actin was used as a reference gene for normalization of methylation levels. Primers for *CDO1*,* TAC1*,* HOXA7*,* HOXA9*,* SOX17*, and *ZFP42* were used. We have previously demonstrated that methylation of these six genes has a high sensitivity and specificity for non-small cell lung cancer (NSCLC) [8, 9].

### Statistical analysis

Continuous variables were summarized using median (interquartile range, IQR) and categorical variables with frequency of events (%). Group comparisons were performed using non-paired wise Wilcoxon rank sum test for continuous variables and Fisher’s exact test for categorical variables. Spearman correlation analysis was used to assess the correlation between continuous variables. Two-sided statistical tests were used. Kaplan Meier curves with log-rank tests were used to compare overall survival between groups. Association with survival was quantified using hazard ratios (HRs) and 95% confidence intervals (CIs) assessed with univariate and multivariate Cox proportional hazard models. The nominal significance level was set at *p* = 0.05. Statistical analysis was performed using R statistical software, version 4.2.2 [16].

As a sensitivity analysis, we conducted univariate age-adjusted Cox proportional hazard models comparing choice of geographic reference (i.e., state-specific vs. U.S.-standardized NDI indices) or definition of NDI (i.e., Messer [18] vs. Powell-Wiley [19, 20] based on Roux and Mair [21]), and we did not observe major differences (Supplemental Table 1).

## Results

### Patient characteristics

A total of 173 patients with non small cell lung cancer met inclusion/exclusion criteria. Across all participants, the median age was 64 years old, with 53% females, 45% White individuals, 43% Black individuals, with a median of 33 pack-year smoking history, 57% with a former smoking history, 24% current smoking individuals, 20% never smoking persons, median tumor size of 2.5 cm, 52% Stage I, 11% Stage II, 16% Stage III and 21% Stage IV (Table 1). Among Black participants, there were 22 (29%) current smokers, 47 (63%) former smokers, and 6 (8%) who had never smoked. In contrast, among White participants, there were 15 (19%) current smokers, 46 (59%) former smokers, and 17 (22%) who had never smoked (*p* = 0.04). The median pack-year smoked by Black participants was 40 (IQR 30–50), compared to 35 pack-years (IQR 12–48) among White participants (*p* = 0.04). A total of *n* = 85 (49%) and *n* = 88 (51%) participants were in the low-deprivation group and high-deprivation group, respectively. Baseline characteristics were similar among the two NDI groups, with no statistically significant differences.

**Table 1:** Baseline characteristics of the 173 non small cell lung cancer patients. Low deprivation is defined as the 1st, 2nd, or 3rd quartiles of the Neighborhood Deprivation Index (NDI; Messer (18)). High deprivation is defined as the 4th quartile of the NDI (Messer (18)). Cells are N (%) unless otherwise specified

	Low Deprivation (*n* = 85)	High Deprivation (*n* = 88)	*p*-value
**Age at diagnosis** (years)^a^ [median (IQR)]	64 (56–73)	63 (57–69)	0.674
**Sex**			0.172
Female	40 (47%)	51 (58%)	
Male	45 (53%)	37 (42%)	
**Race**			0.103
White	43 (51%)	35 (40%)	
Black	30 (35%)	45 (51%)	
Other	12 (14%)	8 (9%)	
**Pack years**^b^ [median (IQR)]	37 (15–50)	30 (13–50)	0.789
**Smoke status**			0.800
Current	21 (25%)	20 (23%)	
Former	46 (54%)	52 (59%)	
Never	18 (21%)	16 (18%)	
**Tumor size** (cm) [median (IQR)]	2.5 (1.7–4.7)	2.8 (1.9-4.0)	0.684
**NSCLC Histologic subtype**			0.552
Adenocarcinoma	72 (85%)	74 (84%)	
Squamous cell carcinoma	13 (15%)	12 (14%)	
Adenosquamous Carcinoma	0 (0%)	2 (2%)	
**Stage**			0.419
I	49 (58%)	41 (47%)	
II	9 (11%)	10 (11%)	
III	12 (14%)	15 (17%)	
IV	15 (18%)	22 (25%)	

*Abbreviations* IQR = interquartile range

^a^*n* = 18 unknown or missing

^b^*n* = 1 unknown or missing

### Neighborhood deprivation, race, and lung cancer stage

**Fig. 1:**
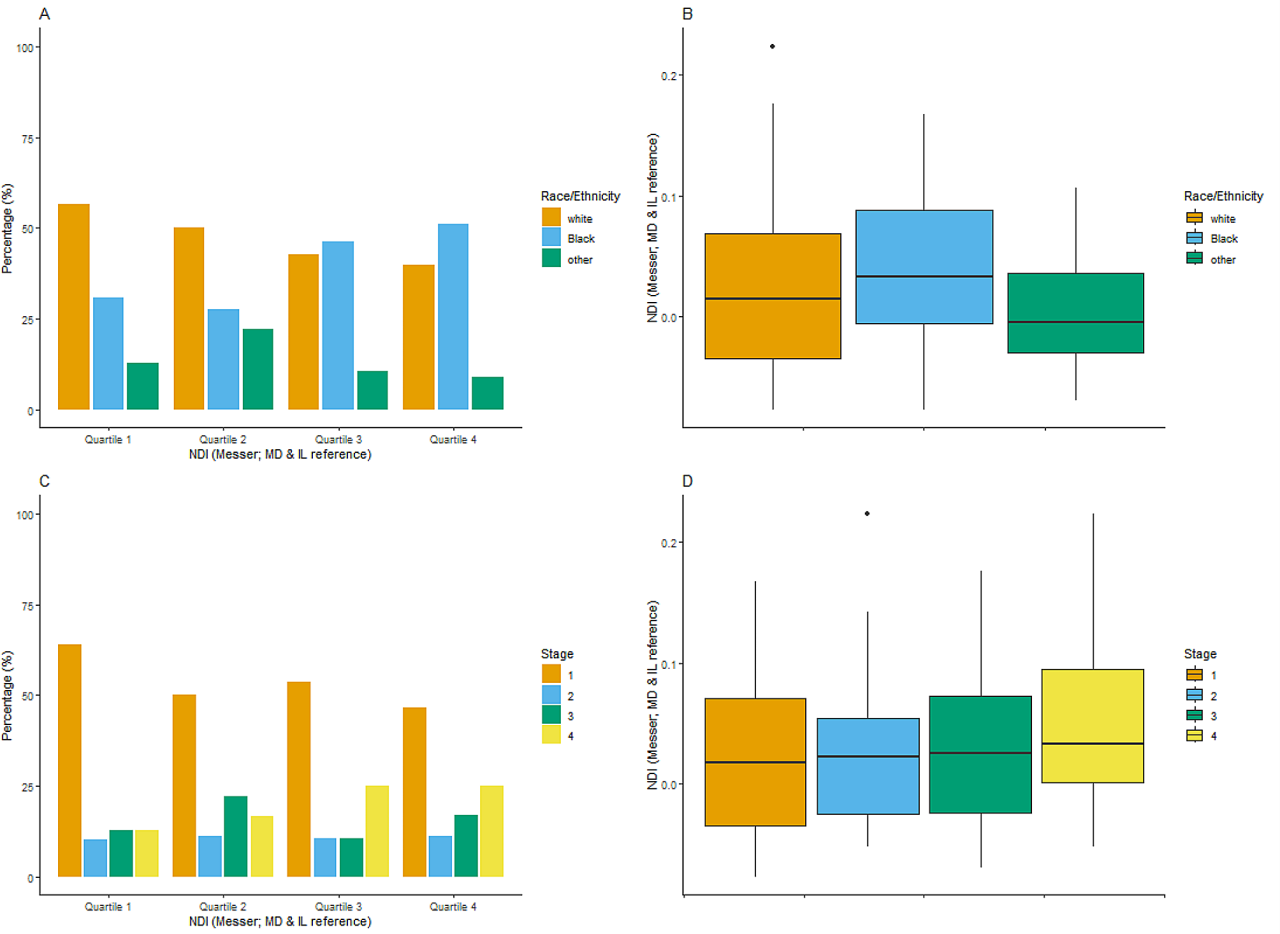
Higher neighborhood deprivation index (NDI) values among Black patients and among patients with late lung cancer stages. **(A)** Proportion of patients by race/ethnicity among the different NDI quartiles. **(B)** Differences in NDI when comparing patients by race/ethnicity. **(C)** Proportion of stage at diagnosis among the different NDI quartiles. **(D)** Differences in NDI when comparing stage at diagnosis

There was a higher proportion of Black patients among 3rd and 4th quartile NDI census tracts and a greater proportion of White or other patients among 1st and 2nd quartile NDI census tracts (*p* = 0.003; Fig. 1A). Median NDI values were significantly higher among Black patients when compared with White patients (*p* = 0.001; Fig. 1B). The proportion of patients with stage I lung cancer was higher among patients living within 1st quartile NDI census tracts, and the proportion of patients with stage IV lung cancer was higher for those in 3rd and 4th quartile NDI census tracts (*p* = 0.001; Fig. 1C). Median NDI showed a trend towards significantly higher values for stage IV lung cancer patients when compared to stage I (*p* = 0.08; Fig. 1D). Higher NDI showed a trend towards significantly association with advanced lung cancer stage OR 1.15 (95%CI:0.99–1.34; *p* = 0.06).

### DNA methylation & neighborhood deprivation

**Fig. 2:**
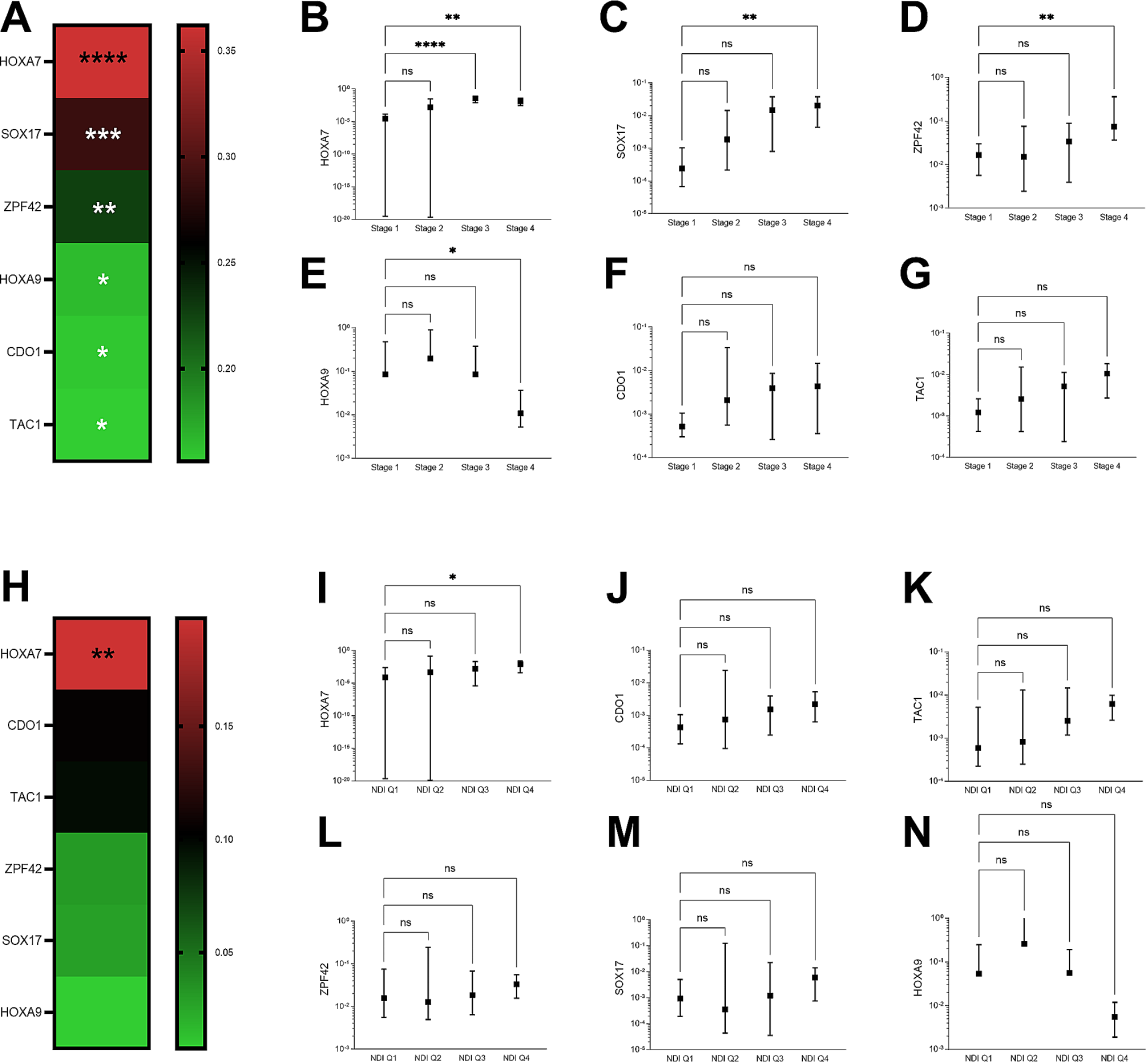
Liquid biopsy epigenetic markers for lung cancer DNA methylation are associated with lung cancer stage and NDI. (**A**) Heatmap of Spearman’s rank correlation coefficient values between lung cancer stage and the DNA methylation for *HOXA7*,* SOX17*,* ZFP42*,* HOXA9*,* CDO1 and TAC1*. (**B-G**) Boxplots showing the differences in median DNA methylation ΔCt values for each of *HOXA7*,* SOX17*,* ZFP42*,* HOXA9*,* CDO1 and TAC1*genes when comparing stages, respectively. (**H**) Heatmap of Spearman’s rank correlation coefficient values between NDI and the DNA methylation for *HOXA7*,* CDO1*,* TAC1*,* ZFP42*,* SOX17*, and *HOXA9*. (**I-N**) Boxplots showing the differences in median DNA methylation ΔCt values for each *HOXA7*,* CDO1*,* TAC1*,* ZFP42*,* SOX17*, and *HOXA9*genes when comparing NDI quartiles, respectively. (Significance values: ****, *P* < 0.0001; ***, *P* < 0.001; **, *P* < 0.01; *, *P* < 0.05; ·, *P* < 0.1; ns, nonsignificant (*P* > 0.1))

There was a significant positive correlation between DNA methylation obtained from liquid biopsies and stage for HOXA7, SOX17, ZFP42, HOXA9, CDO1 and TAC1 (Fig. 2A). Stage IV biopsies had significantly higher DNA methylation than stage I for *HOXA7* (*p* = 0.0001), *SOX17* (*p* = 0.0009) and *ZFP42* (*p* = 0.0007) (Fig. 2B-D). *HOXA9* had significantly lower DNA methylation with stage IV than stage I (*p* = 0.03) (Fig. 2E). When looking at the correlation between DNA methylation and NDI, only *HOXA7* DNA methylation was positively correlated with NDI (*p* = 0.009; Fig. 2H). Patients in 4th quartile NDI census tracts had significantly higher *HOXA7* DNA methylation when compared to the remaining NDI quartiles (*p* = 0.02; Fig. 2I).

### Neighborhood deprivation & survival

**Fig. 3:**
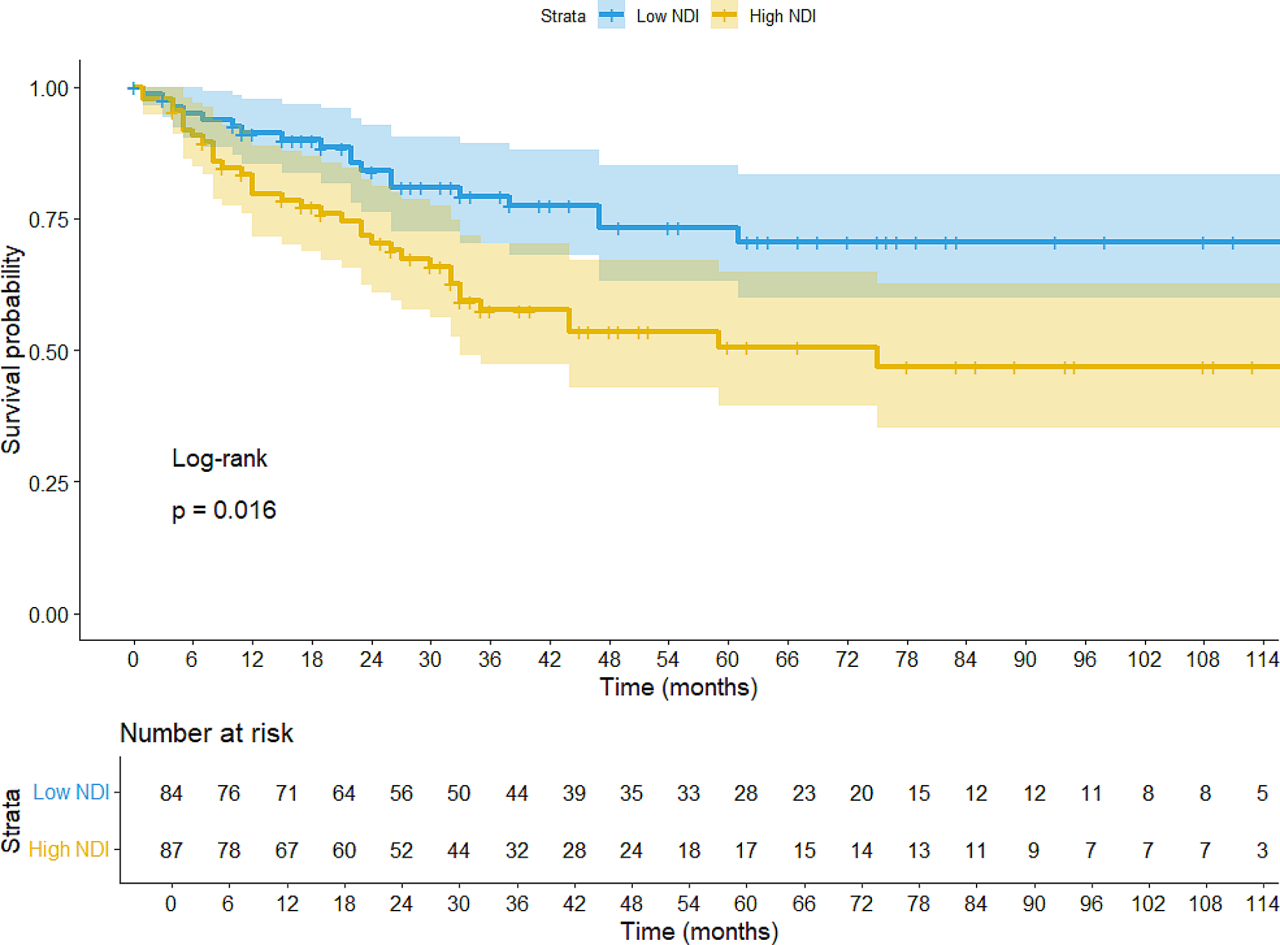
High NDI is associated with shorter survival. Kaplan Meier curve comparing overall survival for high-deprivation group (4th quartile NDI) vs. low-deprivation group (1st, 2nd, and 3rd quartiles NDI)

Median overall survival was significantly shorter for the high-deprivation census tract (4th quartile NDI) with 75 months when compared to the low-deprivation census tract (1st, 2nd, and 3rd quartiles NDI) with 181 months (*p* = 0.02; Fig. 3). The 1-, 2- and 5-year survival rates for the high-deprivation census tract (4th quartile NDI) were 80, 70, and 50%, respectively, compared to 91, 84, and 73% in low-deprivation census tract (1st, 2nd, and 3rd quartiles NDI), respectively.

**Fig. 4:**
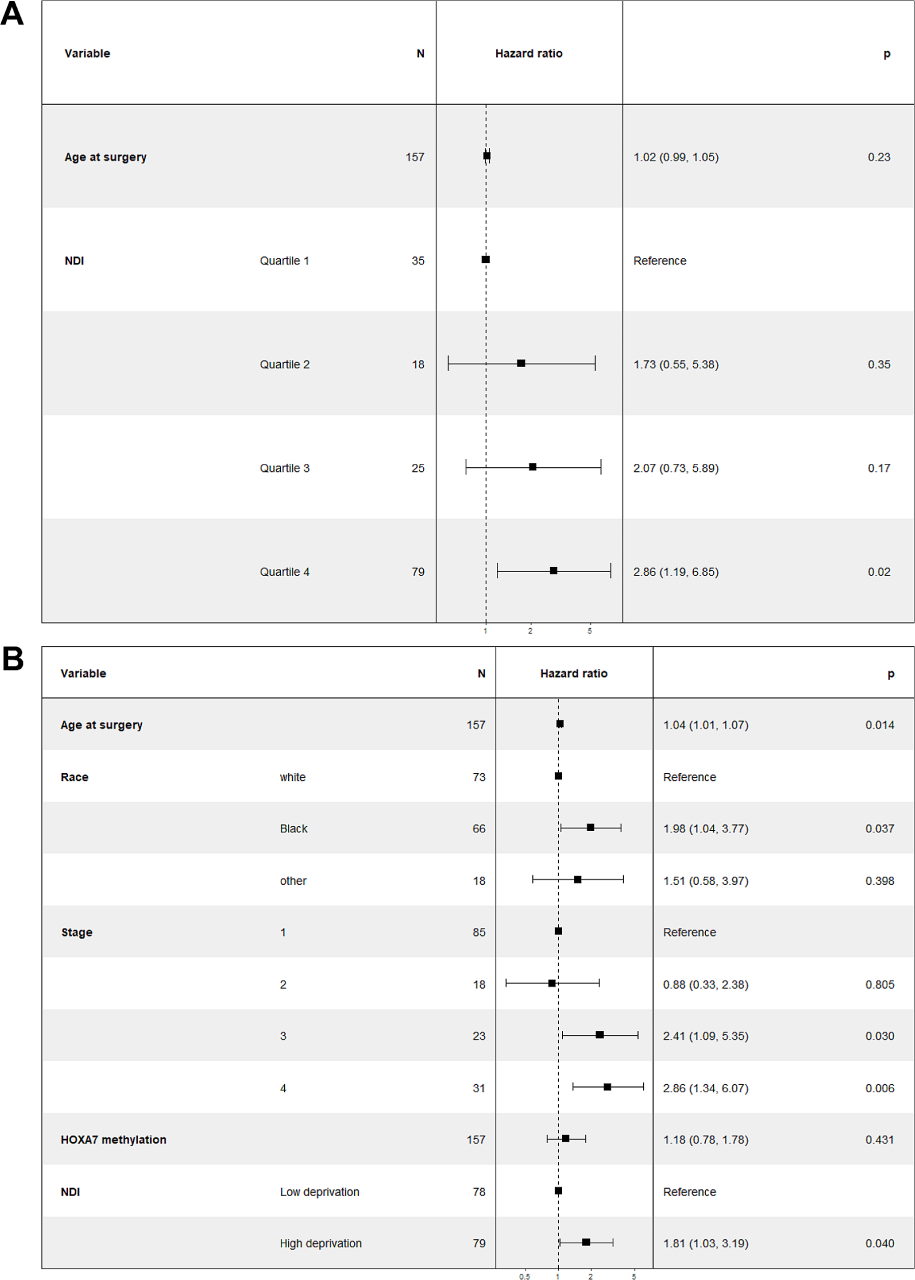
High NDI is associated with increased risk of mortality among lung cancer patients. **(A)** Results from Cox regressions adjusted for age. **(B)** Results from Cox regressions adjusted for age, race, stage, and DNA methylation status

After adjusting for age, living in a 4th quartile NDI census tract was significantly associated with increased mortality with an HR of 2.86 (95%CI: 1.19–6.85; *p* = 0.02; Fig. 4A) compared to living in a 1st quartile NDI census tract. After adjusting for age, race, stage, and DNA methylation status, living in a high-deprivation census tract (4th quartile NDI) remained associated with a higher mortality with an HR of 1.81 (95%CI:1.03–3.19; *p* = 0.04; Fig. 4B) when compared to living in a low-deprivation census tract (1st, 2nd, and 3rd quartiles NDI).

## Discussion

This study suggests that there may be an association between increasing neighborhood deprivation liquid biopsy DNA methylation, shorter survival, and increased mortality risk. Our epigenetic study is unique in that we quantified neighborhood deprivation defined by Messer et al. [18] a validated 8-factor-based index of socioeconomic disadvantage that combines income, educational level, employment status, and housing in a neighborhood (i.e., census tract), constructed from U.S census data, from two U.S metropolitan regions [19, 20]. Previous epigenetic investigations have observed increased DNA methylation [22] and epigenetic age acceleration [23] in those living in areas with higher deprivation defined by Kind et al. [24], and shorter telomere length was observed in participants of the 1999–2002 National Health and Nutrition Examination Surveys in neighborhoods of high deprivation defined by Roux & Mair [21, 25]. All three mentioned definitions of deprivation use information from surveys conducted by the U.S. Census Bureau and are likely correlated. Indeed, we did not observe large differences between neighborhood deprivation defined by Messer et al. [18] and Roux & Mair [21, 25] in a sensitivity analysis (Supplemental Table 1).

In contrast to many previous studies, ours was conducted in a country without a universal health care system, and we found a greater risk for lung cancer mortality in neighborhoods with greater socioeconomic deprivation relative to countries with access to universal healthcare [26, 27]. This suggests how access to care may shape outcomes within the U.S. employer-based health care system. One of the cities in which this study was conducted, Chicago, is known for having relatively high-income inequality (Gini index of 0.5335) and racial segregation [28]. Previous studies have reported that Black individuals have a higher mortality rate from lung cancer despite a lower amount of smoking, as only 8% of Black individuals who smoke cigarettes report heavy smoking (i.e., at least 25 cigarettes per day), as compared with 28% of White individuals [11, 12]. We found that lung cancer mortality was higher in Black individuals (Supplemental Fig. 1) and that neighborhood deprivation may play an integral role in this phenomenon as there was a greater proportion of Black patients in census tracts with the highest deprivation and a greater proportion of White patients in the census tracts with the least deprivation.

The precise mechanisms by which neighborhood deprivation promotes worse outcomes in lung cancer mortality are incompletely characterized; however, several possibilities exist. The Social Determinants Framework for Cancer Health Equity states that health-related disparities stem from social-structural factors [29]. Multi-level factors, including structural inequities, institutional environments, and living environments, are upstream conditions that create health inequities and consequent disparities in cancer. Neighborhood deprivation measures the economic, physical, social, and service environment that individuals live in, all of which impact healthcare access and outcomes. NDI, therefore, is a surrogate for access to care, which can serve as a significant barrier to receiving optimal care and encompasses health insurance, financial barriers, and physical barriers such as transportation. If a long distance is required to receive treatment and/or an individual does not have access to transportation, this can hinder preventative care, such as lung cancer screening, leading to cancer detection at a later stage. Additionally, the ability to regularly visit an infusion center for anti-cancer therapeutics or radiation therapy can lead to decreased efficacy and, hence, poorer outcomes. Discrimination and mistrust in the medical system amongst minority populations, such as Black individuals, may lead to delays in diagnosis and deter participation in clinical trials [13, 14]. Financial toxicity is a considerable barrier in oncologic care [30, 31]. Even if an individual has access to transportation, additional costs such as gas and parking can make cancer care financially infeasible. Only 54% of NCI-designated cancer centers have free parking available for chemotherapy appointments [32]. Furthermore, individuals from disadvantaged neighborhoods may rely more heavily on work income, leading to financial hardships if unable to work. Additionally, individuals may struggle to find necessary childcare to allow for optimal cancer care. Treatment cost itself represents a momentous financial hurdle. Recent studies estimated that the monthly out-of-pocket expenses for the general cancer population were between $316–741 [30, 31]. Additionally, at six months of follow-up, more than 25% of patients require using personal savings, borrowing from friends or family, changing housing, and selling personal assets, and 18% of patients could not afford basic needs [31]. New lung cancer treatments showed improved survival rates but come with a notable financial burden. Disparities persist, with younger, poorer, non-White patients with private insurance less likely to enroll [33]. Participation can lead to financial strain due to insurance issues, travel costs, lost wages, and lodging for follow-up visits.

The impact of deprivation on psychosocial distress within a neighborhood may also play a critical role in cancer mortality. Many neighborhoods with high deprivation have increased violent crime rates, which can, directly and indirectly, exacerbate psychosocial distress by increasing isolation, decreasing access to safe forms of exercise, and reducing access to healthy food [34]. Low-moderate intensity exercise has been shown to help with cancer-related fatigue, nausea, and pain experienced during active treatment [35]. Without safe access to exercise, individuals from disadvantaged neighborhoods will miss these benefits. Psychosocial stressors in cancer have been shown to promote inflammation and oxidative stress, decreased immune surveillance, and activation of the hypothalamic-pituitary-axis, [36] which may lead to increased cancer mortality. Increased prolonged dysregulation of the stress response, also known as allostatic load, is associated with worse survival in patients with cancer. [37] Furthermore, psychosocial distress may exacerbate tobacco smoking, alcoholism, and other substance use disorders, further augmenting adverse outcomes in lung cancer [38, 39].

Epigenetic modifications include promoter hypermethylation, chromatin remodeling, and microRNA expression and are known to drive biological aggressiveness in lung cancer. Social epigenomics is emerging as a biological mechanism by which socio-environmental factors influence health outcomes and disparities [28]. Epigenetic changes, such as promoter hypermethylation, can be influenced by diet, physical activity, psychosocial stress, and environmental exposures, which may be unique within particular neighborhoods. For instance, exposures to heavy metals such as cadmium and particulate matter in air pollution have been associated with epigenetic changes that promote tumorigenesis [40–42]. Several studies have found associations between socioeconomic status and epigenetic changes, including research on aging, depression, inflammation, atherosclerosis, and racial disparities [43–50]. In this current study, we found that *HOXA7* DNA methylation was correlated with higher NDI, and methylation of HOXA7, SOX17, ZFP42, HOXA9, CDO1 and TAC1was associated with advanced stage disease in lung cancer.

There are a few limitations to our study. First, the inherent drawbacks associated with a retrospective study design and the scope of our study were limited by including patients only from two major U.S. cities alone. The sample size of our cohorts was another limitation that has potentially hindered the ability of some of our results to reach statistical significance. Furthermore, our study was limited to a specific set of genes associated with lung cancer. As a result, we were unable to investigate the effects of neighborhood deprivation on the entire epigenome scale. Additionally, since the DNA methylation of the six genes we studied is limited to the promoter gene region, we cannot assume that epigenetic changes occurring on different neighborhood deprivation levels are limited to the promoter gene regions. Our patient cohort was skewed towards earlier-stage disease compared to the incidence stage of lung cancer in the general population. A greater effect may be seen if more patients with metastatic disease were included, given the increased number of visits required relative to early-stage disease and disparities in clinical trial enrollment. According to the Cancer Statistics 2024 report from the American Cancer Society (ACS), [1] the proportion of lung cancer patients diagnosed at lower stages is indeed lower than what we observed in our cohort. In our study, patients were enrolled from two distinct institutions: Johns Hopkins School of Medicine and the University of Illinois Health at Chicago. Each of these institutions provided dedicated clinical coordinators who actively approached patients at oncology clinics at the time of diagnosis to facilitate their participation in our research study. We believe that this proactive approach made patients more receptive to their healthcare providers and navigators, leading to earlier detection of lung cancer within this population. This could explain the higher proportion of stage I diagnoses in our study compared to the ACS report. Lastly, further research is warranted to investigate the impact of neighborhood deprivation on epigenomic changes, with the ultimate goal of reducing disparities in lung cancer. While the exact mechanism remains unclear, it is plausible that the hypermethylation could be driven by the envioronmental effect on overexpression of DNA methyltransferases (DNMTs). This is supported by a growing body of evidence showing the impact of ecosystem, lifestyle, and social environment on DNA methylation [51–54]. Future studies are needed to better understand how disparities in neighborhood deprivation impact DNA methylation. Such research could inform strategies to reduce or prevent this effect.

The findings derived from this study could lead to establishing guidelines for the use of molecular markers in cancer detection, diagnosis, treatment, and monitoring. Biomarkers have the potential to prevent avoidable healthcare costs by providing consistent and accessible liquid-biopsy based screening practices across healthcare settings. Public health strategies can utilize molecular markers to enhance cancer prevention and early detection programs, effectively identifying high-risk individuals through accessible epigenetic testing and providing targeted interventions. Crucially, policy decisions on molecular testing and targeted therapies are essential for ensuring equitable access to underserved populations, thereby enhancing outcomes for these patients regardless of socioeconomic status or geographic location.

## Conclusions

Increased neighborhood-level deprivation may be associated with liquid biopsy DNA methylation, shorter survival, and increased mortality. Changes in health care policy that account for community socioeconomic deprivation may enable more equitable improvement of overall lung cancer mortality.

### Electronic supplementary material

Below is the link to the electronic supplementary material.

Supplementary Material 1

Supplementary Material 2

## Data Availability

Data and materials availability statement: Data generated in this study are available upon request from the corresponding author. The code used to conduct this study analysis is publicly available at: https://github.com/idblr/geomethylation.

## Abbreviations

CDO1: Cysteine dioxygenase type 1
CI: Confidence intervals
CT: Computed Tomography
DNA: Deoxyribonucleic acid
HOXA7: Homeobox protein Hox-A7
HOXA9: Homeobox protein Hox-A9
HR: Hazard ratios
NCI: National Cancer Institute
NDI: Neighborhood deprivation index
NSCLC: Non-small cell lung cancer
SOX17: SRY-box transcription factor 17
STROBE: Strengthening the reporting of observational studies in epidemiology
TAC1: Tachykinin precursor 1
ZFP42: Zinc finger protein 42
