# Supplementary material for: Neighborhood-level deprivation and survival in lung cancer
Source: BMC Cancer. 2024 Aug 6;24:959. doi: 10.1186/s12885-024-12720-w (PMC11301857; doi:10.1186/s12885-024-12720-w)
Supplement: Supplementary file 1 — Supplementary Material 1 [file 12885_2024_12720_MOESM1_ESM.docx]

### Supplemental Table 1 – Comparison of neighborhood deprivation indices and their association with overall cancer survival (N=173) in separate univariate age-adjusted Cox proportional hazard models. Cells are hazard ratios and their 95% confidence intervals.

| NDI Definition | Messer | | Powell-Wiley | |
| --- | --- | --- | --- | --- |
| Reference tracts | Illinois & Maryland | United States | Illinois & Maryland | United States |
| Q1 | Ref | Ref | Ref | Ref |
| Q2 | 1.73 (0.55-5.38) | 2.28 (0.80-6.56) | 2.10 (0.67-6.65) | 2.44 (0.85-7.04) |
| Q3 | 2.07 (0.72-5.90) | 1.97 (0.74-5.24) | 2.31 (0.78-6.85) | 2.53 (0.91-7.01) |
| Q4 | 2.85 (1.29-6.85) | 2.96 (1.29-6.78) | 1.79 (0.61-5.25) | 1.54 (0.60-3.96) |
| Q5 | - | - | 2.97 (1.12-7.84) | 2.67 (1.13-6.33) |
| Low ^a^ | Ref | Ref | Ref | Ref |
| High ^b^ | 1.87 (1.08-3.24) | 1.86 (1.09-3.19) | 1.69 (0.99-2.90) | 1.64 (0.95-2.83) |
| Continuous ^c^ | 0.98 (0.72-1.32) | 1.01 (0.78-1.32) | 1.18 (0.98-1.41) | 1.18 (0.95-1.45) |

^a^ Defined as 1^st^, 2^nd^, and 3^rd^ quartiles for NDI (Messer) and 1^st^, 2^nd^, 3^rd^, and 4^th^ quintiles for NDI (Powell-Wiley)

^b^ Defined as 4^th^ quartile for NDI (Messer) and 5^th^ quintile for NDI (Powell-Wiley)

^c^ NDI (Messer) values are natural log-transformed
