# Supplementary figures and images for: Neighborhood-level deprivation and survival in lung cancer
Source: BMC Cancer. 2024 Aug 6;24:959. doi: 10.1186/s12885-024-12720-w (PMC11301857; doi:10.1186/s12885-024-12720-w)

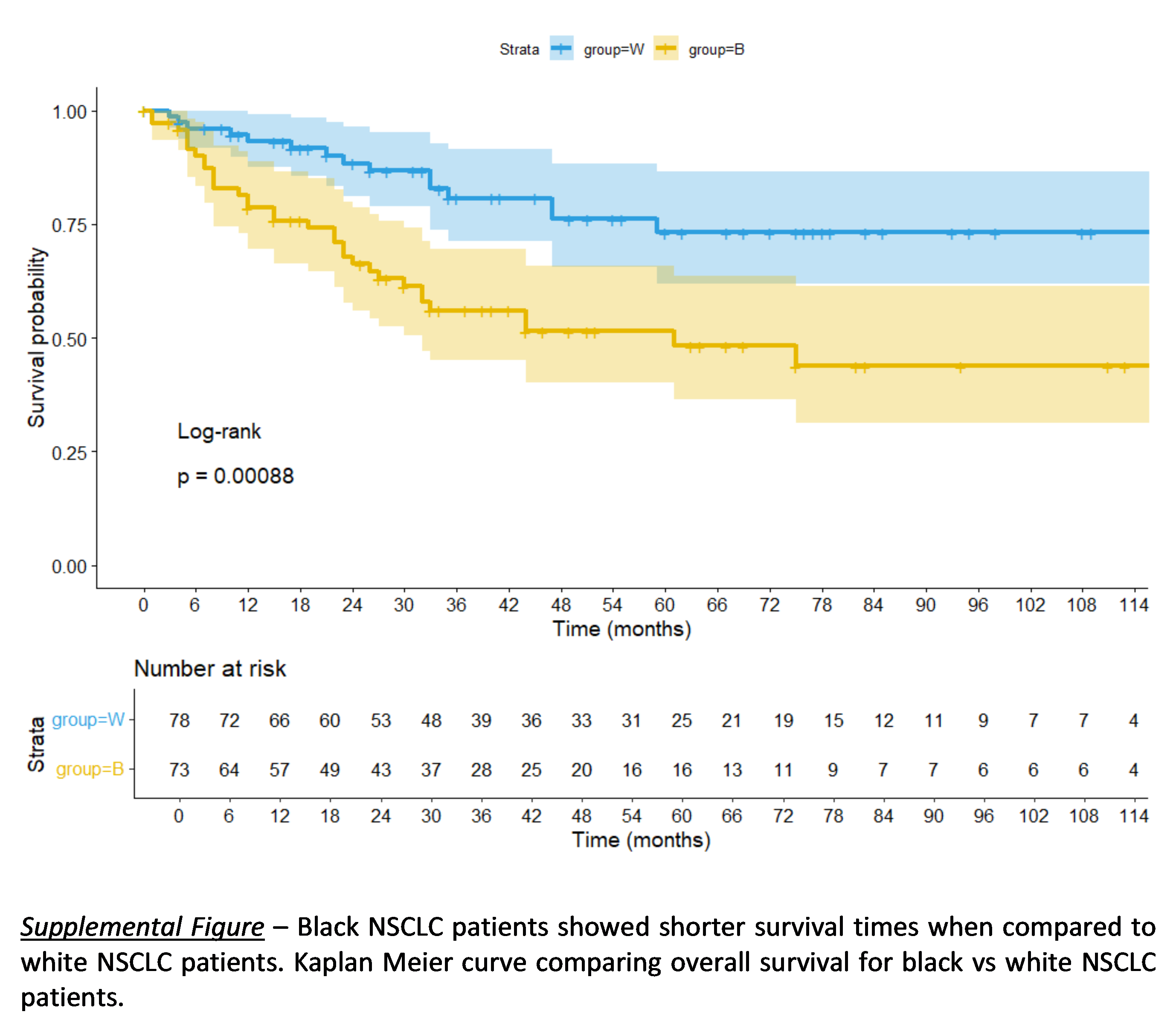

Supplement: Supplementary file 2 — Supplementary Material 2 [file 12885_2024_12720_MOESM2_ESM.tif]
